# Viroids and Viroid-like Circular RNAs: Do They Descend from Primordial Replicators?

**DOI:** 10.3390/life12010103

**Published:** 2022-01-12

**Authors:** Benjamin D. Lee, Eugene V. Koonin

**Affiliations:** 1 National Center for Biotechnology Information, National Library of Medicine, National Institutes of Health, Bethesda, MD 20894, USA; benjamin.lee@nih.gov; 2 Nuffield Department of Medicine, University of Oxford, Oxford OX3 7BN, UK

**Keywords:** viroids, ribozyviruses, primordial replicators, ribozymes, origin of life

## Abstract

Viroids are a unique class of plant pathogens that consist of small circular RNA molecules, between 220 and 450 nucleotides in size. Viroids encode no proteins and are the smallest known infectious agents. Viroids replicate via the rolling circle mechanism, producing multimeric intermediates which are cleaved to unit length either by ribozymes formed from both polarities of the viroid genomic RNA or by coopted host RNAses. Many viroid-like small circular RNAs are satellites of plant RNA viruses. Ribozyviruses, represented by human hepatitis delta virus, are larger viroid-like circular RNAs that additionally encode the viral nucleocapsid protein. It has been proposed that viroids are direct descendants of primordial RNA replicons that were present in the hypothetical RNA world. We argue, however, that much later origin of viroids, possibly, from recently discovered mobile genetic elements known as retrozymes, is a far more parsimonious evolutionary scenario. Nevertheless, viroids and viroid-like circular RNAs are minimal replicators that are likely to be close to the theoretical lower limit of replicator size and arguably comprise the paradigm for replicator emergence. Thus, although viroid-like replicators are unlikely to be direct descendants of primordial RNA replicators, the study of the diversity and evolution of these ultimate genetic parasites can yield insights into the earliest stages of the evolution of life.

## 1. Introduction

The leading current scenario for the origin of life involves the stage of a primordial RNA world, in which RNA molecules are postulated to have doubled in the roles of genomes (replicators) and catalysts (enzymes, or in this case, ribozymes) [1,2,3]. The key ribozymes in the RNA world, obviously, would have been RNA-dependent RNA polymerases (RdRP) that would catalyze their own replication as well as replication of other RNA molecules [4,5]. The major properties of RNA world replicators are easy to surmise: these would be small, stable RNA molecules endowed with the RdRP or capable of recruiting ribozyme RdRP in trans. Obviously, such replicators would not code for any proteins. The phenotype of these primitive replicators would be chemically identical to their genotype.

**Figure 1:**
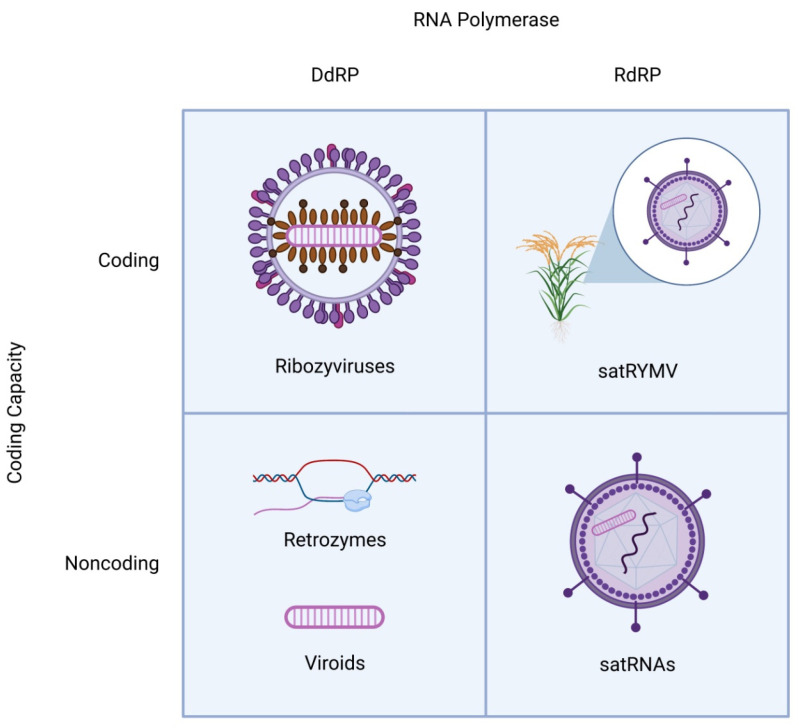
Schematic structures of distinct classes of viroid-like RNAs. All viroid-like agents are divided into four groups depending on whether they encode proteins and are replicated by DNA-dependent or RNA-dependent RNA polymerase. The satRNAs (lower right quadrant) are encapsidated by the helper virus capsid proteins. In one case (top right quadrant), a satRNA of rice yellow mottle virus (satRYMV) appears to encode a protein.

Can such a gene-free genome exist? Viroids, small circular satellite RNAs (satRNAs) and several other groups of similar RNA elements prove that such genomes can and do exist (Figure 1) [6,7,8,9,10]. However, there are no known ribozyme RdRPs. Instead, to replicate without relying on encoded proteins, these agents coopt host enzymes although some of them catalyze certain steps of their own replication using ribozymes formed by the genomic RNA itself. All viroid-like genomes (Table 1) are small, covalently closed circular RNAs (cccRNAs), or at least, go through a cccRNA phase in their replication cycle, and replicate when a polymerase repeatedly rolls around the RNA circle to produce multiple copies, in a process known as rolling circle replication (RCR) [11,12]. The known diversity of these autonomous circular RNAs is growing, with representatives spanning a broad range of genetic elements, from viruses to retrotransposons [13,14,15]. This viroid-like “brotherhood” [14] attracts considerable attention, both because of the importance of some viroid-like agents for human health and agriculture [16], but also because their origin(s) remains enigmatic. Due to their unusual properties, viroids have long been suspected to be relics of the RNA world [17,18,19].

Viroids were the first subviral agents to be discovered and remain the most diverse group in the brotherhood. First described by Diener in 1969 [20,21], these agents are unencapsidated cccRNAs 200–400 nt in length that can cause diseases in plants, some of these fatal. To date, no viroid has been conclusively demonstrated to cause infection in any organisms outside of plants although some can replicate in yeast [22] and may even infect phytopathogenic fungi [23]. Viroid RNAs exhibit extensive self-complementarity resulting in compact, robust, rod-like structures [24,25]. Viroids as well as ribozyviruses replicate via the RCR mechanism, in which the circular RNA is reiteratively transcribed by hijacked host DNA-dependent RNA polymerase (DdRP) into multimeric intermediates of the opposite polarity [12,26,27,28,29,30]. These intermediates are then transcribed themselves and cleaved to unit length, after which the original circular positive-polarity sequence is regenerated via ligation by a co-opted host ligase. The cleavage step varies between the two families of viroids. In members of the *Pospiviroidae* family, a host RNAse cleaves the intermediates, whereas in members of the family *Avsunviroidae*, cleavage of the intermediate is catalyzed by an autocatalytic RNA ribozyme formed by the viroid RNA itself [11,12,29]. Viroids of the *Avsunviroidae* engage in a symmetric process, in which intermediates are ligated into circular forms of the opposite polarity before undergoing the same RCR process again, to produce circular RNAs of the original polarity [31,32]. This process requires viroids to contain two ribozymes (one per RNA strand of each polarity). In members of the *Pospiviroidae* family, only one RCR cycle occurs to create the negative strand [26]. The linear, multimeric negative strand is transcribed, and the positive polarity transcript is cleaved to unit length by a host RNAse. The two families of viroids also vary in their choice of the ligase: members of *Pospiviroidae* use host DNA ligase 1 that they repurpose as RNA ligase [33], whereas members of *Avsunviroidae*, which replicate within plastids, use chloroplast tRNA ligase [34].

Broadly similar to viroids in terms of structure, replication, and range, satRNAs differ in that they are encapsidated, albeit by a helper virus rather than by proteins they encode themselves [10,35]. Furthermore, satRNAs do not rely on host DdRP for replication, but instead employ the RNA-dependent RNA polymerase (RdRP) of the helper virus [12,36]. Additionally, of note, satRNAs vary in their replication mechanisms, beyond using a different polymerase, in that they do not always contain one ribozyme per strand polarity and, when they do, each strand can contain a distinct type of ribozyme [36]. Given that viroids and satRNAs share so many features and are present in the same types of hosts, these agents are thought to share a common ancestor. Phylogenetic reconstructions have shown evidence of such an evolutionary relationship although the exact nature of the common ancestor remains unclear [37].

Neither is the range of viroid-like RNA agents limited to plants nor are they all strictly non-coding. Members of the realm *Ribozyviria*, of which the only well-characterized one is human hepatitis delta virus (HDV), infect animals and encode a single protein that undergoes post-translational modification, producing two distinct forms that perform various functions in virus reproduction including the role of nucleocapsid inside the virions [38,39]. Apart from this distinction, ribozyviruses exhibit the key features of viroids and satRNAs. They too replicate via the rolling circle mechanism catalyzed by host DdRP and employ a virus-encoded ribozyme to cleave the replication intermediates [40,41,42]. HDV, a human pathogen that uses hepatitis B virus as its helper, was the first ribozyvirus to be discovered, but recent metagenomics studies resulted in the identification of multiple ribozyviruses that apparently infect diverse animals [43,44,45,46]. Similarly to satRNAs, ribozyviruses use different types of ribozymes, one of which is unique to members of *Ribozyviria* [47].

Where did the diminutive viroid-like replicators come from? While conclusive evidence is lacking, hypotheses abound, more or less, mirroring the main scenarios considered for the origin of viruses: origin from the pre-cellular replicator pool, regression from existing replicators, and escape from existing genomes [48]. However, the pre-cellular origin hypothesis has a twist in the case of viroids. Unlike the virus-first pre-cellular origin scenario, which postulates that viruses evolved from pre-cellular replicators, the viroid version holds that viroids are not only direct descendants of pre-cellular replicators but also holdovers from the pre-protein RNA world as evidenced by their use of ribozymes for (some stages of) replication [17]. Later, ribozymes would again provide the engine for the transition to the RNA-protein world by serving as the catalytic core of the ribosome. In some of these “viroid early” scenarios, viroids or viroid-like RNAs come across not only as relics from the RNA world but also the first RNA world replicators [19]. Hence, potential special interest of viroid-like agents as a unique window into the hypothetical pre-cellular RNA world. In this article, we discuss the evidence pro and contra the primordial status of viroids, coming to the conclusion that these agents are likely to have much more recent origins, but nevertheless, can offer valuable clues to the features of minimal replicators, even including pre-cellular ones.

## 2. Viroids: Ancient Relics or Recently Emerged Parasites?

Whether all viroid-like agents are monophyletic remains an open question although parsimony suggests a common ancestry [49]. The attributes of the putative common ancestor are hard to pin down in detail but, given that viroids are both the simplest and most autonomous agents within the brotherhood, it seems likely that this ancestor was either viroid-like or satRNA-like [50]. Indeed, the hypothesis on the emergence of these agents within the RNA world is predicated on that assumption. The alternative possibility, that the ancestor was a ribozy-like virus that gave rise to viroids through reductive evolution, is clearly less parsimonious given the higher complexity and apparent narrow spread of ribozyvirus among hosts compared to viroids.

**Figure 2:**
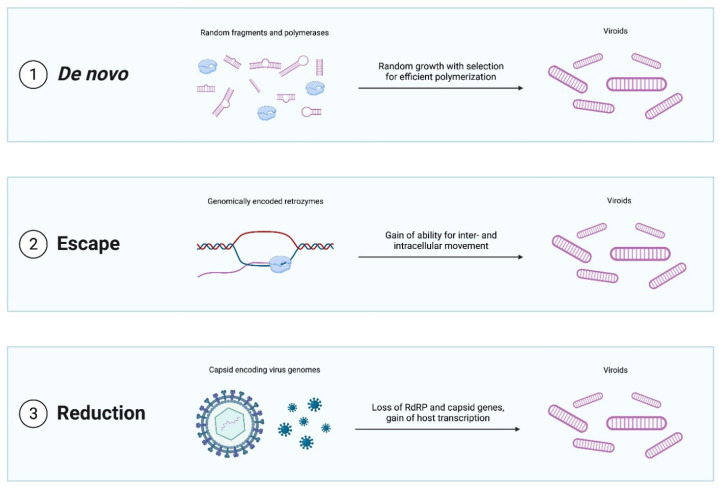
Evolutionary scenarios for the emergence of viroid-like replicators.

A broad variety of possible scenarios for viroid emergence have been proposed (Figure 2). The most radical idea is that viroids emerged de novo [17]. Hard evidence for this mechanism is scant and difficult to obtain although both computational modeling [51] and in vitro experiments [52] indicate that RNAs with properties similar to those of viroids might be produced de novo. Specifically, simulations suggest that rod-shaped and branched circular structures can form when short random RNA sequences fold into hairpins, which then catalyze ligation with other random hairpins, finally producing circular structures. These cccRNAs could then grow in length by both recombination and random insertions. More importantly, such RNAs have the potential to evolve such that sequences with greater stability, robustness, and polymerase affinity being selected for. Thus, Catalan and colleagues [51] conclude that de novo emergence of viroids through a stepwise evolutionary process seeded by any of the diverse small RNAs present in cells, such as microRNAs, is plausible.

Experimental evidence bears a similar picture, albeit with a different replication mechanism. Inspired by the role of DdRPs in viroid and ribozyvirus replication, Jain and colleagues [52] demonstrated that high concentrations of T7 phage DdRP can generate populations of replicating structured linear RNAs from DNA seeds in vitro. Unlike the standard model of viroid replication, which is based on RCR, with monomers ligating to form antigenomic cccRNAs, the mechanism these replicators used bypasses ligation altogether: instead, the polymerase jumps from the 5′ end of the template back to the 3′ end. Recent research also demonstrates that viroid-like self-complementary cccRNAs are readily formed in autocatalytic RNA reaction networks [53]. These lines of research suggest that replicators with the structural hallmarks of viroids could potentially emerge de novo, at least in the presence of a suitable polymerase.

Other proposed mechanisms for the emergence of viroids are also of considerable interest when viewed in the broader context of replicator evolution. The genome escape hypothesis posits that viroids are genomic sequences that managed to achieve semi-autonomous replication [54]. Although arguably less exotic than the de novo scenario, this model nonetheless provides an example of the emergence of a replicator from a non-replicator. In this case, potential ancestors of viroids could be, for example, group I self-splicing introns, the simplest of which form viroid-sized cccRNAs after splicing catalyzed by a ribozyme present in the intron itself [55,56].

In the genome reduction model, viroids emerge from pre-existing replicators, such as viruses, by shedding all coding regions and relying entirely on host enzymes. The classic in vitro evolution experiments of Spiegelman and colleagues [57,58,59] clearly demonstrate the phenomenon of viral genome reduction to viroid size by completely eliminating coding capacity. In these paradigmatic experiments, the 3800 nt genome of RNA bacteriophage Qβ ultimately shrank to a 218 nt non-coding RNA (known as “Spiegelman monster”) under selection for replication speed in the presence of an excess of phage RdRP [60]. A similar process could have conceivably occurred in vivo. Genome reduction is a common phenomenon in obligate parasites of all kinds ranging from bacteria and archaea to worms to arthropods [61,62,63]. In some such cases, the genome reduction can be extreme, with the genomes of some obligate parasitic and symbiotic bacteria shrinking more than an order of magnitude, and mitochondrial genomes more than two orders of magnitude [64,65]. Reductive evolution also readily occurs in viruses, in particular, giving rise to defective interfering particles, and to dramatic contraction of virus genomes upon passages in cell cultures [66,67,68]. Thus, it appears entirely plausible that viroids are ultimately reduced viruses. Identification of sequences homologous to viroids within viral or cellular genomes could lend credence to either of these hypotheses.

Of the three principal models of viroid emergence, two implicitly answer the question of when viroids emerged: both the escape and genome reduction hypotheses, by design, require viroids to have emerged in a cellular context. However, what type of cells? Viroids and satRNAs are limited in range to higher plants, whereas ribozyviruses are limited to animals. Assuming that viroids and viroid-like agents share a common ancestry, horizontal transfer between plants and animals could be an explanation [49]. Hard evidence for the direction of the transfer is lacking, but the greater complexity of ribozyviruses compared to viroids and their limited spread in animals, taken together, seem to point towards a likely origin in plants, with acquisition of the protein-coding gene via recombination in an animal host.

Evidence of emergence of viroids in planta seems to be mounting. Recently, retrotransposons containing hammerhead ribozymes (HHR) similar to those present in viroids, satRNAs, and some ribozyviruses were discovered in plant genomes [69,70,71]. These ribozyme-containing retrotransposons, named retrozymes, are not autonomous and apparently depend on the reverse transcriptase activity of Ty3-like retrotransposons. The retrozymes consist of a 300–700 nt non-coding sequence flanked by long terminal repeats (LTRs) and, apart from the presence of the HHR, resemble other small, non-autonomous retrotransposons that are abundant in plants, such as terminal-repeat retrotransposons in miniature (TRIMs) [72] or small LTR retrotransposons (SMARTs) [73]. The retrozymes are actively transcribed, the transcripts are self-cleaved by HHR and form abundant cccRNAs of both polarities [74,75]. Notably, the circularization is catalyzed by chloroplast tRNA ligase as is the case also in avsunviroids. The RNA sequences of retrozymes are predicted to fold into stable, branched structures resembling those of avsunviroids. Even smaller, non-LTR retrozymes of 170–400 nt have been discovered in genomes of diverse invertebrate and invertebrate animals and also shown to form abundant cccRNAs [76]. The broad representation of retrozymes in plant genomes is compatible with the origin of viroids via retrozyme escape in plants. Given their limited (currently known) host range, such escape seems to be the most likely scenario for the emergence of viroids, in accord with the prescient early hypothesis of Diener [53]. Conversely, the animal retrozymes potentially might have independently given rise to ribozyviruses, as an alternative to the horizontal transfer of viroids discussed above. The evolution of viroid-like replicators from retrozymes seems to combine elements of the escape and reduction scenarios, assuming the retrozymes themselves are products of reductive evolution of autonomous retrotransposons.

In contrast, the viroid-first hypothesis seems to face multiple difficulties. Probably the most damning for this scenario is the limited host range of viroid-like agents, especially their apparent absence in bacteria or archaea. Like spliceosomal introns and many other varieties of non-coding RNAs, viroid-like cccRNAs belong to the expanded “new RNA world” of eukaryotic cells [77,78], and thus, their origin most likely postdates the origin of eukaryotes. Beyond this major problem, replication of viroid-like RNAs in the hypothetical RNA world also seems problematic. Indeed, the extensive, robust secondary structures of these cccRNAs are adapted to mimic DNA and so fool DdRPs and, possibly, also to avoid RNAi host response [9]. Neither of these factors that apparently shape the evolution of viroid-like RNAs is relevant in the primordial setting. On the contrary, replication of such highly structured RNAs by the hypothetical ribozyme polymerases would be hampered, even beyond the more generic problems faced by such polymerases despite extensive experimental attempts to evolve accurate and processive ones [4].

Thus, the retrozyme escape hypothesis appears to be the most parsimonious scenario for the origin of viroid-like cccRNAs. Refutation of this hypothesis would imply that retrozymes either are integrated viroids or a case of convergence. Although HHRs appear to have evolved on multiple occasions independently [79], the similarities between retrozymes and viroids are so pronounced that the most likely explanation remains that viroids are escaped retrozymes.

## 3. Viroids as Paragons of Minimal Replicator Emergence

As discussed, the possibility that viroids and other similar cccRNAs are direct descendants of primordial replicators that inhabited the hypothetical ancient RNA world appears to be remote. However, the likely relatively recent origins of viroid-like RNAs cannot deprive them of the status of minimal replicators. It does not appear to be a chance coincidence that the Spiegelman monster, the minimal parasite artificially evolved under conditions when the sole selective factor was the speed of replication, is the same size as the smallest viroids and satRNAs [60]. This size, approximately 200 nt, is likely to be close to the ultimate low bound for a replicator.

Replicators are genetic elements that encode information that is necessary and sufficient for their replication but lack the full complement of genes required for providing energy and building blocks for replication [80,81,82,83,84]. For energy and building blocks, replicators depend on reproducers, biological entities whose reproduction involves physical rather than only informational continuity as in the case of replicators [81,84]. The paradigmatic reproducers are, obviously, cells—*omnis cellula e cellula*, according to Virchow. More precisely, all cellular life forms can be conceived as symbioses of a reproducer and a replicator, the genome [85]. Cellular genomes are ultimate cooperative replicators, but generally, replicators span wide ranges of replicative autonomy and aggressiveness/cooperativity with respect to the host reproducer [83,86]. 

Some replicators, such as many large viruses or self-synthesizing transposons, encode a (nearly) complete replication and sometimes transcription machinery, and thus possess a high degree of replicative autonomy. Others rely mostly or completely on the host replication and expression apparatus. Viroids are the extreme manifestation of the latter strategy—arguably, the ultimate parasites. Generally, the information content of replicators splits into “replicase expression signal” (RES), which can consist of one or more genes encoding replicative enzyme (s) and possibly various accessory proteins, and “replicase recognition signal” (RRS), non-coding elements that are necessary and sufficient to hijack the host replication (or expression) machinery [87]. Viroids have adopted the ultimate parasitic strategy, namely, selection for maximum efficiency of RRS accompanied by complete elimination of RES, under the reduction scenario or by never acquiring RES, under the de novo scenario. More precisely, this is the evolutionary strategy of pospiviroids, whereas avsunviroids add a notable twist by engaging viroid-embedded ribozymes in some stages of replication. The HHRs appear easy to evolve [79] and could be the simplest evolutionary step towards increased replicative autonomy.

Evolution towards ultimate parasitism occurs already at the stage of retrozymes as well as other non-autonomous transposons that hijack the replication and transposition machinery of autonomous transposons with closely similar RRS. However, retrozymes have only limited replication capacity restricted to copy-paste transposition within the host genome. Viroids likely escaped from the host genomes by evolving the ability to redirect the host DdRP towards viroid RNA replication. Identification of an intermediate on the proposed evolutionary path from retrozymes to viroid-like agents—namely, a retrozyme capable of replicating autonomously within a cell—would be a major step in demonstrating the emergence of viroid-like replicators as an ongoing process. There are some indications that retrozymes might possess that capacity, in particular because multimeric intermediates of both polarities have been detected and the high error rate of retrozymes suggests polymerization by DdRP, but definitive experimental evidence is still lacking [69,76].

Although viroids are unlikely to be relics of the pre-cellular stage of life evolution, they provide a unique window into the origin of parasitic replicators, which most likely occurred on multiple, independent occasions, via retrozyme escape and, possibly, de novo. Similar events could have transpired during the pre-cellular stage of evolution where ultimate parasites similar to viroids with respect to size and reproduction cycle, but not necessarily secondary structure, could have evolved from autonomous replicators. 

## 4. Viroid-like RNAs and Emergence of Protein Coding

If emergence of viroid-like replicators is an ongoing process, there seems to be no reason why some of these replicators would not evolve to encode proteins, and indeed, although neither viroids nor retrozymes encompass protein-coding genes, other members of the viroid brotherhood do. The best studied case, obviously, are the ribozyviruses that encode a single protein of unclear provenance, which is employed for a typical virus function, encapsidation of the genome [38,39]. An unrelated example is a satRNA of rice yellow mottle virus (satRYMV), the smallest known satRNA at 220 nt, that has been shown to encode proteins in overlapping open reading frames, covering most of the genome, and reiteratively translated, in parallel to the replication of satRYMV via reiterative transcription [88]. Translation of satRYMV proceeds through multiple rounds by switching frames and thus yielding proteins of supergenome size. Notably, satRYMV contains ribozymes in both polarities within the protein-coding region. Although too far reaching conclusions, perhaps, should not be drawn from this single case, satRYMV seems to present a remarkable example of de novo emergence of proteins. Search for other similar cases might shed light on the evolution of protein coding in new replicators and, possibly, even at processes that occurred in the primordial RNA world.

More generally, it appears likely that the diversity of protein-coding viroid-like agents is substantially underappreciated as suggested, in particular, by the recent discovery of an expanded range of ribozyviruses [43,44,45,46,47]. Furthermore, it seems likely that evolution of coding and non-coding viroid-like RNAs is a two-way street, with protein-coding genes both gained and lost on multiple occasions. Comprehensive screening of the rapidly growing metagenomic data for different types of such agents should reveal their actual diversity and evolutionary relationships.

## 5. Conclusions

Viroids and viroid-like RNAs are minimal replicators, the only known ones that encode no proteins and probably do not far exceed the theoretical minimum replicator size. Therefore, the idea that these RNA replicators are relics of the hypothetical primordial RNA world appears both straightforward and highly attractive. Yet, this scenario seems to be poorly compatible with the available evidence including both the host range of these agents that currently appears to be limited to multicellular eukaryotes and the properties of the viroid-like RNAs that are clearly attuned to replication by sophisticated protein polymerases. In contrast, a direct line of descent appears to be traceable from retrozymes, non-autonomous retrotransposons, to viroids. It appears likely that viroid-like replicators evolved from retrozymes, possibly, on several independent occasions. However, this likely relatively recent origin hardly diminishes the fundamental interest of viroid-like RNAs as the paradigm for the emergence of new replicons as ultimate parasites, at different stages of life’s evolution, including the pre-cellular stage. The existence of protein-coding viroid-like genomes, those of ribozyviruses and the single so far characterized protein-coding viroid-like satRNA, indicates that there is no impassable gulf between the non-coding, minimal RNA replicators and protein-coding replicators including bon fide viruses. Conceivably, evolutionary transitions between these two types of replicators occurred repeatedly during evolution. The recent major expansion of the diversity of ribozyviruses that until then have been represented solely by human HDV suggests that we are presently unaware of the full range of viroid-like replicators, and even might be missing most of their diversity. Comprehensive search of transcriptomes and metatranscriptomes for viroid-like RNAs using dedicated computational pipelines can be expected to substantially expand their diversity and shed light on their origins and evolutionary relationships.

## Figures and Tables

**Table 1:** The major types of viroid-like cccRNAs.

Viroid-like cccRNAs	Size	Host	Ribozymes	Known Coding Capacity
Viroids	246–450 nt	Plants	HHR when present	None
Ribozyviruses	1547–1735 nt	Metazoans	HDVR or HHR	One conserved protein
Retrozymes	300–1116 nt	Eukaryotic genomes	HHR	None
satRNAs	220–457 nt	Plants	HHR or hairpin	None (except satRYMV)
Hypothetical primordial replicator	~200 nt	None (RNA world)	HHR	None
